# ADME-drug-likeness: enriching molecular foundation models via pharmacokinetics-guided multi-task learning for drug-likeness prediction

**DOI:** 10.1093/bioinformatics/btaf259

**Published:** 2025-07-15

**Authors:** Dongmin Bang, Juyeon Kim, Haerin Song, Sun Kim

**Affiliations:** Interdisciplinary Program in Bioinformatics, Seoul National University, Seoul, 08826, Republic of Korea; AIGENDRUG Co., Ltd, Seoul, 08758, Republic of Korea; Department of Statistics, Seoul National University, Seoul, 08826, Republic of Korea; Interdisciplinary Program in Artificial Intelligence, Seoul National University, Seoul, 08826, Republic of Korea; Interdisciplinary Program in Bioinformatics, Seoul National University, Seoul, 08826, Republic of Korea; AIGENDRUG Co., Ltd, Seoul, 08758, Republic of Korea; Interdisciplinary Program in Artificial Intelligence, Seoul National University, Seoul, 08826, Republic of Korea; Department of Computer Science and Engineering, Seoul National University, Seoul, 08826, Republic of Korea

## Abstract

**Summary:**

Recent breakthroughs in AI-driven generative models enable the rapid design of extensive molecular libraries, creating an urgent need for fast and accurate drug-likeness evaluation. Traditional approaches, however, rely heavily on structural descriptors and overlook pharmacokinetic (PK) factors such as absorption, distribution, metabolism, and excretion (ADME). Furthermore, existing deep-learning models neglect the complex interdependencies among ADME tasks, which play a pivotal role in determining clinical viability. We introduce ADME-DL (drug likeness), a novel two-step pipeline that first enhances diverse range of molecular foundation models (MFMs) via sequential ADME multi-task learning. By enforcing an A→D→M→E flow—grounded in a data-driven task dependency analysis that aligns with established PK principles—our method more accurately encodes PK information into the learned embedding space. In Step 2, the resulting ADME-informed embeddings are leveraged for drug-likeness classification, distinguishing approved drugs from negative sets drawn from chemical libraries. Through comprehensive experiments, our sequential ADME multi-task learning achieves up to +2.4% improvement over state-of-the-art baselines, and enhancing performance across tested MFMs by up to +18.2%. Case studies with clinically annotated drugs validate that respecting the PK hierarchy produces more relevant predictions, reflecting drug discovery phases. These findings underscore the potential of ADME-DL to significantly enhance the early-stage filtering of candidate molecules, bridging the gap between purely structural screening methods and PK-aware modeling.

**Availability and implementation:**

The source code for ADME-DL is available at https://github.com/eugenebang/ADME-DL.

## 1 Introduction

One of the critical steps in early-stage drug discovery is in effectively filtering out candidates with poor potential for approval, thereby reducing research costs and timelines (Zhu *et al.* 2023). Recent advancements in AI-driven generative models have intensified this challenge by producing massive virtual libraries, creating thousands per hour. While this technological progress opens a new frontier for drug discovery, it also places extensive pressure on downstream evaluation to prioritize drug-like leads with speed and accuracy.

Traditionally, drug-likeness evaluation focused more on rule-based scoring schemes rather than precisely classifying molecules either as “drug-like” or “non-drug-like.” Extending from Rule of Five by Lipinski *et al.* (1997), these early computational filters rely on molecular weight, lipophilicity, or polar surface area. Quantitative estimate of drug-likeness score (Bickerton *et al.* 2012) extend this idea by generating a continuous score with structural properties. However, due to the inherent ambiguity in setting appropriate thresholds, these approaches lacked a robust mechanism for effectively identifying nondrug molecules.

To analyze large-scale data on approved drugs and investigational compounds in databases such as DrugMAP, PubChem, and ZINC, machine-learning (ML)-based methods emerged as a more sophisticated alternative. These approaches framed drug-likeness as a binary classification problem, leveraging algorithms like Support Vector Machines, Bayesian Neural Networks, and Random Forests to learn from molecular descriptors and fingerprints (Zhu *et al.* 2023). While these models demonstrated improvements in prediction performance, their effectiveness are limited by their reliance only on structural representations of molecules.

More recently, advancements in ML have further refined drug-likeness prediction through modeling complex representations. Techniques such as autoencoders and graph neural networks (GNNs) have gained attention for their ability to capture structural and topological information (Sun *et al.* 2022, Zhu *et al.* 2023). Furthermore, there have been attempts to resolve the ambiguity in defining negative sets or “non-drug-like” molecules, via unsupervised learning or Positive-Unlabeled (PU)-learning frameworks (Lee *et al.* 2022).

While these methodologies have advanced drug-likeness prediction significantly, they predominantly focus on structural information, often ignoring pharmacokinetic (PK) aspects (absorption, distribution, metabolism, excretion; ADME) that are fundamental to a drug’s clinical performance (Zhu *et al.* 2023). Drugs’ PK characteristics are closely associated with stability, bioavailability, which are critical factors for evaluating drug safety and efficacy. Hence, filtering molecules only based on structural rules or similarities does not guarantee they will exhibit the necessary ADME properties for *in vivo* success.

Furthermore, even when ADME data are considered, existing methods often treat these properties as independent or use them in simplistic ways (Fu *et al.* 2024, Gu *et al.* 2024). In reality, ADME processes are highly interdependent—absorption can affect distribution, which in turn influences metabolism and excretion. Modeling these “task dependencies” is essential for capturing the PK-guided flow of a compound and thus enhancing the predictive power of drug-likeness prediction models.

To overcome the two main limitations—(1) the reliance on purely structural features for drug-likeness, and (2) the lack of modeling inter-relationships among ADME components—we propose ADME-DL, a drug-likeness prediction framework that integrates PK data guided by data-driven analysis and established pharmacological knowledge.

We first pretrain molecular foundation models (MFMs) (ranging from GNNs to Transformers) on 21 ADME endpoints to construct an ADME-informed embedding space. Unlike conventional models that rely solely on structural descriptors, this ADME-informed embedding reflects the intricate biological and PK factors that contribute to a compound’s clinical success. This embedding space serves as the foundation for the multi-layer perceptron (MLP)-based drug-likeness classifier, offering improved accuracy and interpretability.

Specifically, we enforce a sequential (A→D→M→E) order across the ADME tasks to reflect the natural PK flow and model the fundamental lifecycle of drugs within the body (Ernstmeyer and Christman 2023). Such multi-task learning (MTL) approach is, to the best of our knowledge, the first to ground its design on a data-driven ADME task-dependency graph that strongly aligns with established PK principles. By allowing upstream tasks (e.g. absorption) to inform downstream tasks (e.g. metabolism), our method reduces conflicting signals and preserves biological context, enhancing accuracy and interpretability.

Experimental results on three benchmark sets demonstrate that our ADME-based embedding outperforms existing structure-focused methods. Importantly, the sequential MTL framework is model-agnostic, delivering performance gains across four different MFMs. Ablation studies and a detailed task-dependency graph reveal clear synergies among the ADME endpoints, underscoring on how their interplay enhances classification accuracy. Practical relevance is confirmed through case studies on clinically annotated drugs, with the predicted drug-likeness scores correlating with drug discovery stages. By leveraging the ADME task-dependency based modeling, our framework overcomes the limitations of purely structural approaches, offering a more comprehensive and pharmacologically informed method for early-stage screening of therapeutic compounds.

## 2 Materials and methods

**Figure 1.**
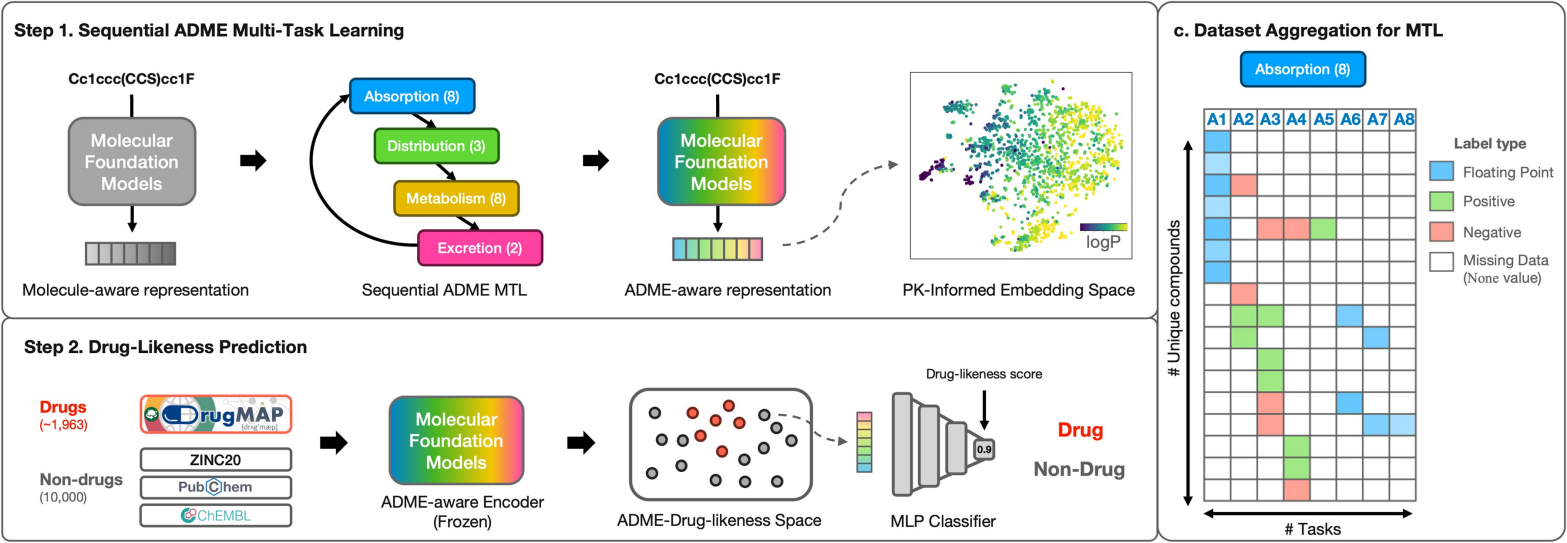
Overview of our PKs-aware sequential MTL and drug-likeness prediction framework. (a) Step 1: sequential ADME MTL trains MFMs, such as GNNs and Transformers, on 21 endpoints using a biologically guided A→D→M→E sequence. This approach respects task dependencies and reflects the natural PK flow, resulting in a PK-informed embedding space enriched with ADME context. (b) Step 2: Using the pretrained ADME-aware MFMs, drugs and nondrugs are encoded into the ADME-Drug-Likeness embedding space. An MLP classifier is then applied to accurately distinguish drugs from nondrugs. This model-agnostic framework improves drug-likeness prediction by integrating PK principles with molecular representations. (c) For dataset aggregation of the tasks for each ADME category, overlapping compounds between sets are merged, and assigned None value to missing data.

**Figure 2.**
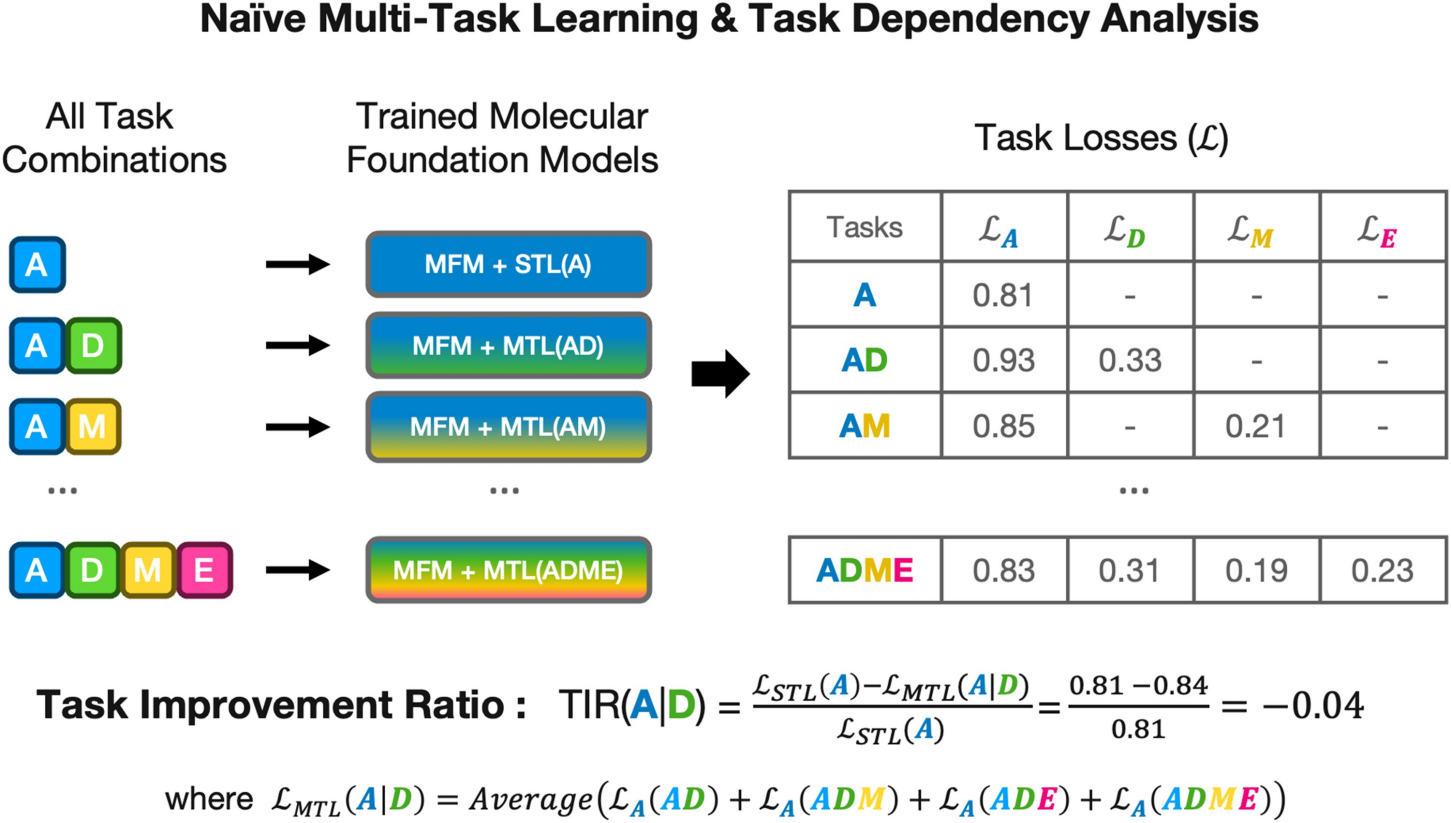
Naïve MTL and task dependency analysis. Using the naïve MTL strategy, we trained all possible task combinations of the ADME endpoints on the MFM (GraphMVP) and recorded the task losses for each configuration. From this task loss table, we computed the task improvement ratio. This metric quantifies how co-training with other tasks (e.g. D) influences the performance of a target task (e.g. A). The resulting task dependency graph reveals the relationships between ADME tasks.

Our framework, ADME-DL (Drug-likeness), leverages MFMs through Sequential ADME MTL for enhanced drug-likeness prediction (Fig. 1). The ADME-DL framework operates in two steps: first, sequential ADME-MTL is applied to the MFM $M_{\text{PT}}$ to learn embeddings for 21 ADME tasks, creating a pharmacokinetically enriched embedding space *z*. Second, an MLP classifier C is trained on the embeddings to distinguish approved drugs from large-scale chemical libraries, ensuring both enhanced predictive accuracy and alignment with biologically motivated workflows. The proposed Sequential ADME MTL is based on a novel PK ADME task dependency analysis that is used to produce a biologically grounded foundation for ADME (Fig. 2). The overview of training ADME-DL and corresponding sections are organized in Algorithm 1.

Algorithm 1 Overview of ADME-DL Framework

**Inputs:**

 ADME endpoint dataset $D_{\text{ADME}}$ and

drug/non-drug dataset $D_{\text{DL}}$  →  *Section 1*

**Initialize:**

 Pretrained MFM $M_{\text{PT}}$  →  *Section 2*

Drug-likeness MLP Classifier C

**Step 1:** Sequential ADME MTL →  *Section 3*

Define ADME task groups: *A*, *D*, *M*, *E* from $D_{\text{ADME}}$

**for** epoch e=1 to *E* **do**

 **for**category in [A→D→M→E]  **do**

  Train $M_{\text{PT}}$ on category tasks

  Resolve intra-category gradient conflicts

 **end for**

**end for**

Obtain $M_{\text{ADME}}\leftarrow \text{Train}(M_{\text{PT}},D_{\text{ADME}})$

**Step 2:** Drug-Likeness Prediction →  *Section 4*

Encode molecules from $D_{\text{DL}}$ into z using $M_{\text{ADME}}$

Train classifier C on z to predict drug/non-drug labels

**Return**  C and z

### 2.1 Datasets

#### 2.1.1 ADME datasets

ADME properties are fundamental PK parameters that determine the concentration and behavior of a therapeutic compound after administration. Understanding ADME is critical for effective drug design and optimization, as it influences both efficacy and safety profiles.

However they are abstract concepts, and require specific experimental endpoints to quantitatively measure their properties in practice. For this study, we leveraged the Therapeutic Data Commons (Huang *et al.* 2021), an open-source collection of curated datasets, which provides 21 endpoints that map to the four ADME categories.

Table 1 summarizes each endpoint’s dataset size, label distribution, and basic descriptive statistics. Endpoints for Absorption mainly measure membrane permeability or transporter interaction, while Distribution measures organ-associated coefficients. Metabolism specifically focuses on enzyme-substrate interactions of CYP450 enzymes, and Excretion measures drug elimination process.

**Table 1.** Statistics of 21 ADME-associated endpoint datasets provided in the Therapeutics Data Commons database.

Dataset	Category	No. of data	Task type
Caco-2 permeability	Absorption	906	Regression
PAMPA permeability	Absorption	2177	Classification
Human intestinal absorption (HIA)	Absorption	578	Classification
P-glycoprotein (Pgp) substrate	Absorption	1212	Classification
Bioavailability	Absorption	640	Classification
Lipophilicity	Absorption	4200	Regression
Solubility	Absorption	9982	Regression
Hydration free energy	Absorption	642	Regression
BBB (blood–brain barrier)	Distribution	1975	Classification
PPBR	Distribution	1614	Regression
VDss	Distribution	1130	Regression
CYP P450 2C19 Inhibition	Metabolism	12 665	Classification
CYP P450 2D6 Inhibition	Metabolism	13 130	Classification
CYP P450 3A4 Inhibition	Metabolism	12 328	Classification
CYP P450 1A2 Inhibition	Metabolism	12 579	Classification
CYP P450 2C9 Inhibition	Metabolism	12 092	Classification
CYP2C9 Substrate	Metabolism	666	Classification
CYP2D6 Substrate	Metabolism	664	Classification
CYP3A4 Substrate	Metabolism	667	Classification
Half life	Excretion	667	Regression
Human hepatocyte clearance	Excretion	2122	Regression

##### 2.1.1.1 Dataset aggregation

To enable both naïve MTL and sequential MTL by PK categories, we implemented a dataset aggregation pipeline to aggregate the 21 ADME endpoints (Fig. 1c). Overlapping compounds were merged by consolidating their labels, while missing endpoint data were labeled as None and excluded from training and evaluation for those tasks. Additionally, to harmonize continuous and categorical endpoints, regression targets were normalized on train set distribution, ensuring balanced loss contributions across tasks. This preprocessing framework allowed efficient and consistent optimization across diverse ADME objectives. For sequential MTL, endpoints were grouped into four categories (Absorption, Distribution, Metabolism, Excretion), while naïve MTL combined all endpoints into a single dataset. Further details are provided in the Supplementary Methods section.

#### 2.1.2 Drug-likeness prediction datasets

Drug-likeness prediction task aims to classify compounds as drugs or nondrugs to aid in identifying potential drug candidates, where positive sets consist of approved drugs, and negative sets are derived from large chemical libraries of nonapproved compounds.

We obtained information on approved drugs from the DrugMAP database (Li *et al.* 2023), which contains both small-molecule and biologically derived therapeutics. This set covers a broad distribution of molecular sizes and scaffolds, reflecting real-world chemical diversity within approved therapies.

We curated three distinct datasets as negative sets to ensure diverse and challenging nondrug examples. ZINC20 (Irwin *et al.* 2020) is a large-scale virtual screening library containing chemically diverse early-stage candidates. PubChem (Kim *et al.* 2016) is an open-access database with a wide range of structurally heterogeneous molecules. ChEMBL (Gaulton *et al.* 2012) consists of bioactive, drug-like compounds, including many investigational molecules that have not received regulatory approval. Initial similarity analyses among the four datasets (including DrugMAP) revealed minimal overlap (Supplementary Fig. S1), highlighting the importance of evaluating on multiple negative sets to ensure robustness. To create the benchmark sets, we randomly sampled 10 000 compounds from each of the three datasets. By combining these with DrugMAP’s approved drug data, our dataset design supports comprehensive benchmarking and facilitates reliable performance evaluation of the proposed models.

In total, our drug-likeness benchmark set comprises 1963 approved drugs (from DrugMAP) and 10 000 nondrugs from each of the three external sources (ZINC, PubChem, ChEMBL), forming three distinct datasets: DrugMAP-ZINC, DrugMAP-PubChem, and DrugMAP-ChEMBL. To ensure robust evaluation, we randomly partitioned each combined dataset into five subsets, preserving label distribution, and assigned them to training, validation, and test sets for 5-fold cross-validation.

### 2.2 Molecular foundation models

Our ADME-DL framework is designed to be model-agnostic, allowing integration of various MFMs. MFMs first learn general molecular representations by pretraining on large, unlabeled molecule databases via self-supervised learning, and then refine these representations through supervised learning on smaller, labeled datasets (Méndez-Lucio *et al.* 2024).

We specifically evaluated four distinct molecular encoders, each employing a different strategy for encoding molecules into informative representations *z*:

- **Fingerprint + MLP:** A simple yet effective method representing molecules by binary vectors (fingerprints), indicating presence or absence of specific molecular substructures. Paired with a straightforward neural network (MLP), it serves as a reliable baseline.
- **Two Pretrained GNNs:** GNNs represent molecules as graphs, where atoms are nodes and bonds are edges. They learn molecular representations by aggregating information from neighboring atoms, capturing both structural and chemical properties. Pretrained GNNs, such as GraphMVP (Liu *et al.* 2022) and MolCLR (Wang *et al.* 2022), use self-supervised learning to extract meaningful molecular patterns from large datasets. GraphMVP employs multi-view contrastive learning, aligning 2D topology and 3D conformations into a shared representation. MolCLR, on the other hand, enhances learning by training on multiple augmented views of molecular graphs, improving its ability to generalize across different chemical structures.
- **Pretrained Transformers:** Transformers, originally designed for natural language processing, have been adapted for molecular modeling to capture long-range atom interactions. Molecular Attention Transformer (MAT) (Maziarka *et al.* 2024) applies an attention mechanism to molecular graphs, treating atoms as tokens in a sentence. It undergoes large-scale self-supervised pretraining on over 1 million compounds, using a masked atom prediction task to develop a deep understanding of chemical structures. This enables MAT to effectively model complex molecular relationships and enhance downstream prediction tasks.

All MFMs were patched with MLP prediction heads per endpoint, with all MFM parameters fine-tuned during the ADME-MTL step. Among the MFMs, GraphMVP was selected as the representative model of our study based on its superior performance compared to other models. Details of each model are provided in the Supplementary Methods section.

### 2.3 Sequential MTL

MTL aims to effectively leverage both task-specific and shared information to address multiple related tasks simultaneously. In many real-world scenarios, tasks are not simply independent or arbitrarily grouped; they follow a logical or biological progression, forming a task-dependency or task correlation (Yu *et al.* 2024). The ADME processes exemplify such sequential dependencies, where each PK stage naturally builds on the previous one (Ernstmeyer and Christman 2023). Below, we first describe the standard, naïve MTL setup, then introduce our Sequential Multi-Task Learning framework which explicitly leverages these biological relationships.

#### 2.3.1 Naïve MTL

Traditional MTL frameworks combine multiple tasks into a single training process during each epoch, both regression and classification endpoints are trained simultaneously, and the model computes a cumulative loss (e.g. the sum or average of individual task losses) to perform a single backward pass for updating model parameters. While this strategy is straightforward, it implicitly treats each ADME endpoint as an independent objective. As a result, any inherent biological dependency, such as absorption preceding distribution, remains unrecognized. This can lead to suboptimal performance and reduced interpretability, especially if the underlying tasks have directional or hierarchical relationships.

In our naïve MTL configuration, we aggregated all 21 ADME endpoints into a single multi-labeled dataset.

##### 2.3.1.1 Task dependency analysis

A key limitation of naïve MTL lies in the assumption that all tasks share a homogeneous feature space. When tasks with differing objectives are trained together without accounting for dependencies, conflicting representations may emerge. For instance, a model may neglect subtle absorption signals that are crucial for downstream metabolic predictions, or it may “smooth out” domain-specific signals if they contradict learning objectives for other tasks. This phenomenon, often referred to as task interference, is a well-known phenomenon in MTL and can degrade performance or generalization (Yu *et al.* 2024).

To investigate these conflicts more concretely, we conducted all-pairwise multi-task experiments between A, D, M, and E, analyzing how the performance of a given task changes when co-trained with different tasks. To the best of our knowledge, this is the first in-depth brute-force analysis of task dependencies within the ADME framework, providing novel insights into the interplay between PK components. Specifically, we define a task improvement rate (TIR) motivated from Maninis *et al.* (2019), designed to quantify how multi-task training influences each ADME endpoint compared to its performance under single-task learning (STL):


$$\text{TIR}(A|B)=\frac{L_{\mathrm{STL}}(A)-L_{\mathrm{MTL}}(A|B)}{L_{\mathrm{STL}}(A)}$$

where $L_{\mathrm{STL}}(A)$ is the single-task loss for A, and $L_{\mathrm{MTL}}(A|B)$ is the average loss for A when it is co-trained with B (or B plus other tasks, e.g. AB, ABC, ABD). A negative TIR indicates that MTL with B increases A’s loss (i.e. degrades performance), while a positive rate signifies an improvement (i.e. reduced loss) over the single-task baseline. By examining these rates across all combinations of A, D, M, and E, we gain insights into which ADME tasks synergize or interfere with one another under naïve MTL. Experimental results are discussed in Section 3.2.

By treating each endpoint as an isolated objective, naïve MTL not only misses the biological ordering of processes but also risks corrupting domain-specific signals crucial for drug-likeness predictions. This analysis highlights the need for a more structured approach, such as sequential MTL, which respects the PK flow and resolves conflicts between related tasks.

#### 2.3.2 Sequential multi-task framework

Our sequential multi-task framework is designed to mirror the PK lifecycle of a drug, where ADME naturally occur in sequence. By leveraging the inherent causality which is also reflected in task dependencies, our method allows upstream processes (e.g. absorption) to inform downstream processes (e.g. excretion).

##### 2.3.2.1 Grouping and training strategy

In particular, we grouped the 21 endpoints into four categories based on PK stages of Absorption (A): 8 tasks, Distribution (D): 3 tasks, Metabolism (M): 8 tasks, and Excretion (E): 2 tasks.

Tasks *within* each category are trained in parallel (e.g. MTL across the eight Absorption endpoints), while training *between* categories proceeds sequentially in any user-given order, including A→D→M→E. Furthermore, to resolve the intra-category gradient conflicts, we applied PCGrad (Yu *et al.* 2020) approach to minimize interference among tasks that naturally interact, thereby stabilizing training and enhancing model convergence (see Supplementary Methods).

This structured order ensures that representations learned from earlier stages naturally influence subsequent stages, mirroring the causality between ADME in pharmacological progression. The A–D–M–E cycle is repeated until the total loss from all the endpoints converge.

Overall, our Sequential ADME MTL framework is designed to incorporate PK causality directly into the training loop, rather than *ad hoc* aggregation of tasks and hoping for synergy to emerge implicitly. The combination of task-grouped parallel training and PCGrad-based conflict resolution within each stage ensures stable optimization. This design not only boosts predictive accuracy for individual ADME endpoints but also lends a coherent interpretative layer to the model’s decision-making, culminating in improved drug-likeness classification. Further details in the training scheme are organized in the Supplementary Methods section.

### 2.4 MLP drug-likeness predictor

The MLP classifier C serves as the final step in our framework, distinguishing drugs from nondrugs. The MLP is trained with the ADME-informed embeddings *z* produced by MFM model $M_{\text{ADME}}$, through binary cross-entropy loss until convergence. To ensure consistency and comparability, we employ the same MLP architecture across all embedding spaces, regardless of the encoder used to generate them. We further note that the MLP is trained on top of the extracted embedding vectors, without fine-tuning of MFM parameters. The model’s hyperparameter search space, including the number of layers, hidden dimensions, and learning rates, along with their impact on model performances are detailed in Supplementary Tables S1 and S2. In addition, comparison of MLP classifier with other widely used ML classifiers are organized in Supplementary Table S3.

## 3 Results and discussion

### 3.1 Drug-likeness prediction on benchmark datasets

We evaluated our proposed Sequential ADME MTL approach on three distinct datasets of DrugMAP-PubChem, DrugMAP-ZINC, and DrugMAP-ChEMBL, encompassing over 30 000 molecular compounds collectively. These large-scale negative sets feature high chemical diversity, posing substantial challenges for ML algorithms. We compared our method against classical fingerprint-based classifiers (Random Forest, MLP, Support Vector Machine) and MFMs (MolCLR, MAT, GraphMVP), along with state-of-the-art ML-based drug-likeness predictors [DGCAN (Sun *et al.* 2022), DeepDL (Lee *et al.* 2022), and DBPP-Predictor (Gu *et al.* 2024)]. Table 2 summarizes the results using balanced metrics, including Matthews correlation coefficient (MCC), F1-score (F1), and area under the precision–recall curve (AUPR), accounting for the imbalance between positive and negative labels. Our Sequential MTL framework consistently achieves the highest performance, with up to a +3.4% improvement in F1 compared to DBPP-Predictor in the DrugMAP-ZINC dataset. Notably, the largest gain appears in the DrugMAP-ChEMBL dataset (F1 = 0.347) compared to fingerprint-based approaches (average F1 = 0.094) and other MFMs (average F1 = 0.067). This advantage underscores our method’s robustness and adaptability across challenging real-world chemical spaces. The full performance metrics are provided in Supplementary Tables S4–S6.

**Table 2.** Drug-likeness prediction performances on three drug–nondrug dataset pairs.

	DrugMAP-ZINC			DrugMAP-PubChem			DrugMAP-ChEMBL		
	MCC	F1	AUPRC	MCC	F1	AUPRC	MCC	F1	AUPRC
FP-RF	0.627 (0.0195)	0.578 (0.0225)	0.745 (0.1846)	0.153 (0.0352)	0.135 (0.0309)	0.248 (0.0161)	0.188 (0.0323)	0.134 (0.0237)	0.185 (0.0305)
FP-MLP	0.750 (0.0184)	0.746 (0.0203)	0.816 (0.0180)	0.182 (0.0691)	0.125 (0.0652)	0.287 (0.0262)	0.014 (0.0282)	0.002 (0.0040)	0.183 (0.0146)
FP-SVM	0.819 (0.0097)	0.819 (0.0101)	0.879 (0.0090)	0.219 (0.0340)	0.182 (0.0321)	0.276 (0.0243)	0.207 (0.0166)	0.146 (0.0148)	0.213 (0.0191)
MolCLR	0.512 (0.0209)	0.454 (0.0312)	0.557 (0.0242)	0.175 (0.0323)	0.106 (0.0331)	0.245 (0.0167)	0.081 (0.0168)	0.028 (0.0100)	0.127 (0.0046)
MAT	0.716 (0.0257)	0.710 (0.0256)	0.764 (0.0278)	0.210 (0.0266)	0.147 (0.0268)	0.275 (0.0252)	0.077 (0.0137)	0.026 (0.0048)	0.117 (0.0106)
GraphMVP	0.716 (0.0472)	0.702 (0.0598)	0.788 (0.0312)	0.266 (0.0299)	0.191 (0.0330)	0.322 (0.0207)	0.212 (0.0856)	0.148 (0.0767)	0.246 (0.0271)
DGCAN	0.189 (0.2313)	0.137 (0.2497)	0.112 (0.1631)	0.039 (0.0349)	0.012 (0.0123)	0.023 (0.0031)	−0.000 (0.0004)	0.000 (0.0000)	0.023 (0.0045)
DeepDL	0.467 (0.0240)	0.388 (0.0307)	0.754 (0.0465)	0.185 (0.0247)	0.106 (0.0198)	0.199 (0.0203)	0.102 (0.0217)	0.059 (0.0106)	0.141 (0.0129)
DBPP-Predictor	0.831 (0.0106)	0.824 (0.0126)	0.891 (0.0148)	**0.438 (0.0522)**	0.418 (0.0586)	0.450 (0.0169)	**0.398 (0.0358)**	0.326 (0.0462)	**0.459 (0.0318)**
ADME-DL	**0.850 (0.0107)**	**0.852 (0.0104)**	**0.912 (0.0106)**	0.435 (0.0274)	**0.420 (0.0323)**	**0.463 (0.0232)**	0.389 (0.0267)	**0.347 (0.0416)**	0.380 (0.0327)

Mean and SD of 5-fold cross-validation are provided. Best performances are marked in bold, and second-best underlined.

These consistent improvements confirm that ADME-DL’s biologically aligned multi-task training yields more comprehensive representations of drug-likeness, offering both domain context and predictive power. Our comprehensive evaluation on three distinctive negative sets ranging from ZINC, PubChem to ChEMBL demonstrates the robustness and utility of the ADME-DL framework and its Sequential MTL.

### 3.2 Task-Dependency exploration between ADME

Initially, we performed a pairwise naïve MTL analysis of each ADME component, which served as the foundation for developing our Sequential ADME MTL framework. Specifically, we trained models on every possible combination of these tasks (e.g. AD, AM, AE, ADM, ADE, etc.) without any sequential constraints. This approach allowed us to isolate how each task influences—or is influenced by—other tasks in a purely data-driven manner.

**Figure 3.**
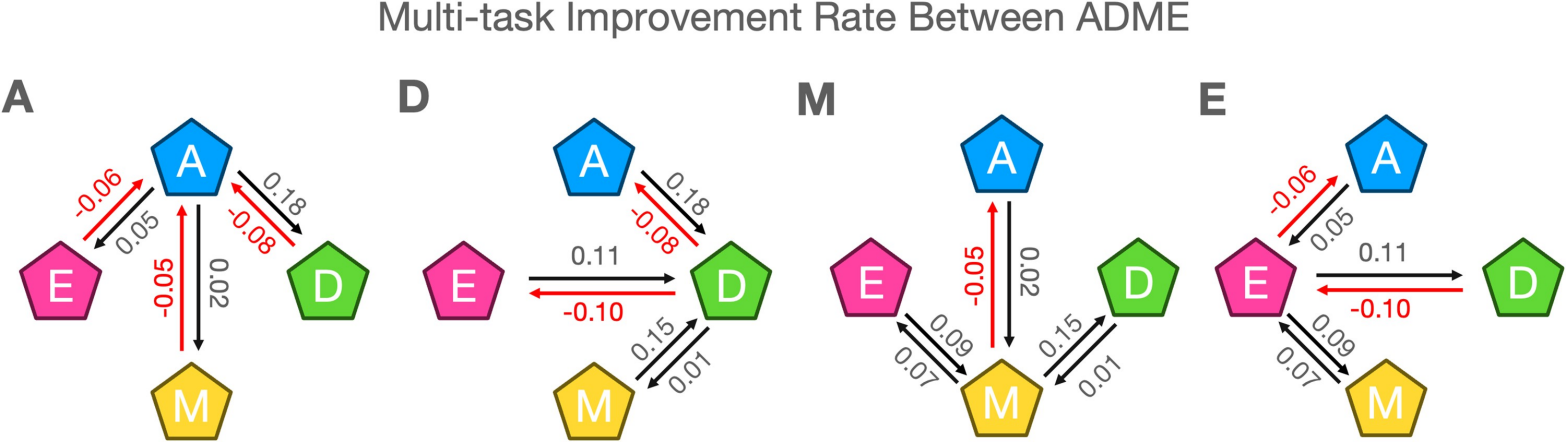
The improvement rate of each task in predicting ADME within a MTL framework. ADME multi-tasking impacts ADME predictions. Notably, task A enhances the predictive performance of D, M, and E tasks, while being interfered from other tasks.

As a result, we observed an interesting asymmetry of task relations between each component (Fig. 3), with full performances organized in Supplementary Tables S7 and S8. The most interesting and clearly observed trend is that while Absorption brings improvement in all other components, it experiences performance decline when co-trained with other components (Fig. 3A). This is in line with the general sequence of absorption preceding distribution, metabolism, and excretion is fundamental to understanding drug behavior in the body (Ernstmeyer and Christman 2023).

Distribution tasks, by contrast, exhibit performance gains when paired with all other tasks (Fig. 3D). This phenomenon mirrors the well-established PK interplay in which blood concentrations depend on absorption levels, metabolism rates, and hepatic clearance (Mansoor and Mahabadi 2023). Moreover, metabolite formation can alter protein-binding profiles, impacting plasma protein binding rates (PPBR) and, consequently, the volume of distribution at steady state (VDss) (Smith *et al.* 2015). Such interdependencies underscore why distribution profits from co-training with the entire ADME pipeline.

Metabolism and Excretion also mutually benefit each other’s performance (Fig. 3M and E), a finding consistent with the fact that the liver, a primary site of Metabolism, also plays a crucial role in Excretion, demonstrating the close relationship between these processes (Teo *et al.* 2015). Furthermore, Metabolism enhances Excretion by increasing water solubility of drugs, making them more easily excreted by the kidneys (Garza *et al.* 2023). However, we observed an opposing effect between Distribution and Excretion, suggesting that while Distribution operates on a more systemic scale (Teo *et al.* 2015), Excretion tasks remain heavily reliant on hepatic clearance (Garza *et al.* 2023). The discrepancy between the systemic nature of Distribution and the organ-specific nature of Excretion may create conflicting representation updates during joint training, leading to performance degradation for Excretion while improvement for Distribution.

Overall, these results highlight how naïve MTL can yield unintended conflicts among hierarchically or mechanistically linked tasks. Our analysis underscores the importance of respecting the PK flow (A→D→M→E) to avoid representational interference and preserve domain-specific signals, laying the data-driven foundation for our sequential multi-task approach.

### 3.3 Model-Agnostic enhancement of ADME-informed MTL

A key objective of this study is to demonstrate the universality of our Sequential ADME MTL strategy, highlighting that the performance gains achieved through ADME sequential learning are consistently observed across diverse models and datasets. To do so, we integrated four distinct molecular encoders—a Transformer-based MAT, two pretrained GNNs (GraphMVP and MolCLR), and a traditional extended-connectivity fingerprint—within our sequential training framework. Table 3 shows that sequential ADME pre-training significantly improves drug-likeness classification compared to both no pre-training and naïve MTL on the DrugMAP-ZINC dataset. For instance, GraphMVP’s MCC rose by an average of +1% over naïve MTL, while MAT achieved a +5.3% gain and the fingerprint-based approach attained +2.6%.

**Table 3.** Drug-likeness prediction performances of four molecular encoders augmented with different ADME pretraining-strategy.

	MCC	Gain	F1	Gain	AUPRC	Gain
GraphMVP	0.72 (0.047)	–	0.70 (0.060)	–	0.79 (0.031)	–
GraphMVP${}_{\text{MTL}}$	0.84 (0.012)	+14.3%	**0.85 (0.012)**	+17.6%	0.91 (0.010)	+13.2%
GraphMVP${}_{\text{EMDA}}$	0.83 (0.019)	+13.3%	0.83 (0.019)	+15.7%	0.89 (0.017)	+11.2%
GraphMVP${}_{\text{ADME}}$	**0.85 (0.011)**	+15.3%	**0.85 (0.010)**	+17.6%	**0.91 (0.011)**	+13.2%
MAT	0.72 (0.026)	–	0.71 (0.026)	–	0.76 (0.028)	–
MAT${}_{\text{MTL}}$	0.72 (0.020)	0%	0.72 (0.020)	+1.4%	0.79 (0.024)	+3.8%
MAT${}_{\text{EMDA}}$	0.69 (0.017)	−4.3%	0.68 (0.019)	−4.4%	0.75 (0.022)	−1.3%
MAT${}_{\text{ADME}}$	**0.76 (0.019)**	+5.3%	**0.76 (0.019)**	+6.6%	**0.82 (0.017)**	+7.3%
FP-MLP	0.75 (0.018)	–	0.75 (0.020)	–	0.82 (0.018)	–
FP-MLP${}_{\text{MTL}}$	0.75 (0.019)	0%	0.75 (0.019)	0%	0.81 (0.010)	−1.2%
FP-MLP${}_{\text{EMDA}}$	0.76 (0.016)	+1.3%	0.75 (0.017)	0%	0.80 (0.012)	−2.5%
FP-MLP${}_{\text{ADME}}$	**0.77 (0.015)**	+2.6%	**0.77 (0.015)**	+2.5%	**0.84 (0.011)**	+2.4%
MolCLR	0.51 (0.021)	–	0.45 (0.031)	–	0.56 (0.024)	–
MolCLR${}_{\text{MTL}}$	0.55 (0.023)	+7.3%	0.52 (0.022)	+13.5%	**0.58 (0.020)**	+3.4%
MolCLR${}_{\text{EMDA}}$	0.53 (0.028)	+3.8%	0.49 (0.034)	+8.2%	0.53 (0.017)	−5.7%
MolCLR${}_{\text{ADME}}$	**0.57 (0.022)**	+10.5%	**0.55 (0.024)**	+18.2%	0.57 (0.023)	+1.8%

Mean and SD of 5-fold cross-validation are provided, along with performance gain ratio compared to vanilla models. Best performance per molecular encoders are marked in bold. (Encoder${}_{\text{MTL}}$: Naïve MTL, Encoder${}_{\text{ADME}}$: Sequential MTL in A–D–M–E order.).

While naïve MTL and Sequential MTL in A→D→M→E order brought consistent performance increase compared to the vanilla MFMs, models trained with Sequential ADME MTL displayed the highest performances for each case. Conversely, reverse-Sequential MTL (E→M→D→A) occasionally underperformed naïve MTL on certain encoders (MAT, MolCLR), suggesting that training in an order that contradicts the normal PK flow can create conflicting gradient updates or misaligned representations, particularly in architectures that rely heavily on integrated contextual information. Full results including DrugMAP-PubChem and DrugMAP-ChEMBL datasets (Supplementary Tables S9–S11) further reinforce the consistent trend of performance improvement through Sequential ADME MTL, along with occasional performance decline with naïve or reverse-Sequential MTL. Additional analysis with the main backbone MFM, GraphMVP, using mutated orders of D→E→A→M and M→A→E→D further validated the effectiveness of the PK-informed A→D→M→E sequential MTL, depicted in Supplementary Table S12.

To ensure the robustness of our framework, we examined whether redundancy between compounds from the pretraining (ADME) and fine-tuning (drug-likeness) datasets artificially inflated performance. We conducted an experiment by removing molecules highly similar to those in the fine-tuning sets from the ADME dataset before pretraining (Supplementary Fig. S2). Results showed no significant change in classification performance across models, suggesting that the observed improvements stem from PK-guided sequential learning, rather than dataset overlap. Detailed results are provided in Supplementary Table S13.

Overall, these findings highlight the model-agnostic strength of our sequential framework, which aligns the training process with the natural ADME sequence. By leveraging data-driven task dependencies observed among ADME endpoints that align with real-world pharmacological processes, our framework enriches the learning context for drug-likeness prediction, enabling more accurate and biologically interpretable results. To note, based on its better performances, we adopted GraphMVP as the representative MFM of ADME-DL and utilized them for further case studies.

### 3.4 Case studies

#### 3.4.1 Evaluation of sequential ADME training on clinically annotated compounds

Beyond quantitative evaluation, we examined a subset of clinically relevant drugs with established PK profiles to further validate the reliability of our sequential approach (Table 4). Specifically, we examined five drugs—warfarin, atorvastatin, omeprazole, phenytoin, and ibuprofen—selected for their well-documented ADME profiles as annotated by domain experts. In particular, warfarin is associated with CYP2C9 metabolism, atorvastatin with CYP3A4, and omeprazole with CYP2C19, whereas phenytoin and ibuprofen both exhibit high PPBRs. As summarized in Table 4, Sequential ADME MTL yielded accurate ADME property predictions (e.g. correct enzyme specificity or PPBR thresholds) and consistently assigned higher drug-likeness scores to these compounds, aligning with established PK knowledge. In contrast, the STL baseline incorrectly classified atorvastatin and omeprazole as nondrugs (drug-likeness score <0.5) and showed reduced accuracy in characterizing metabolism and plasma binding properties. Notably, our sequential approach achieved this enhanced performance without any misclassification of these approved drugs, underscoring its ability to capture critical ADME dependencies that ultimately translate into more reliable drug-likeness predictions.

**Table 4.** Comparison of predicted ADME property values, and drug-likeness scores for clinically well-annotated drugs using Sequential ADME Training and STL.

Drug	Clinical Annotation	Model	Prediction	Drug-likeness
Wafarin	CYP2C9 = 1	SeqADME	**0.5236**	**0.9989**
	(Wadelius and Pirmohamed 2007)	STL	0.3076	0.6507
Atorvastatin	CYP3A4 = 1	SeqADME	**0.6097**	**1.000**
	(Bottorff 2006)	STL	0.2578	0.0046
Omeprazol	CYP2C19 = 1	SeqADME	**0.8533**	**0.8214**
	(Zanger and Schwab 2013)	STL	0.7159	0.1836
Phenytoin	PPBR > 90	SeqADME	**0.8568**	**0.9656**
	(August *et al.* 1997)	STL	0.6612	0.9242
Ibuprofen	PPBR ≃ 98	SeqADME	**0.6757**	**0.9966**
	(August *et al.* 1997)	STL	0.5122	0.9539

Predictions from Sequential ADME (SeqADME) Training align more closely with domain expert-annotated labels for individual ADME properties and overall drug-likeness scores, demonstrating improved accuracy and interpretability compared to the STL baseline. The highest values per each drug are marked in bold.

#### 3.4.2 ADME-DL’s drug-likeness scores reflect the progress of drug discovery

**Figure 4.**
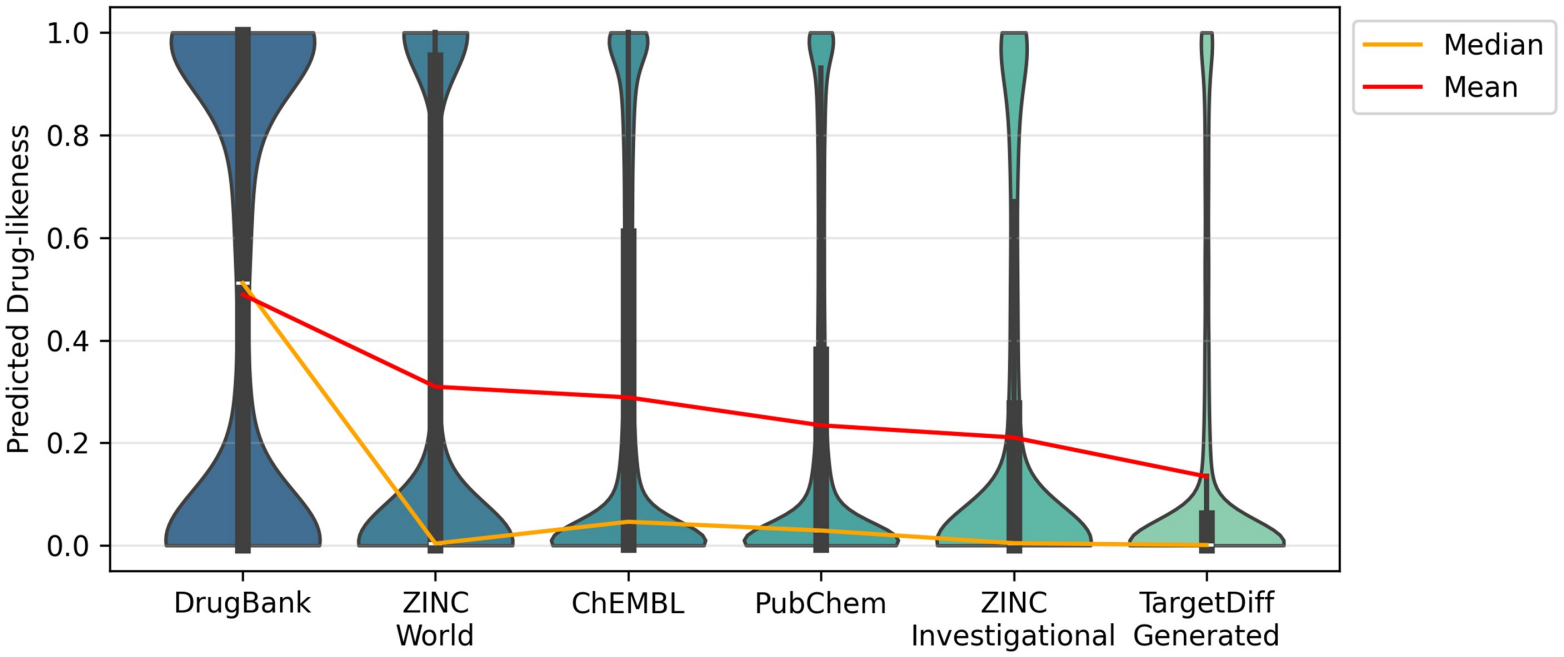
Distribution of drug-likeness score by ADME-DL on compounds in diverse drug discovery phases. The mean and median values of drug-likeness scores decrease from approved drugs to investigational and lastly AI-generated compounds, reflecting progress of drug discovery.

To validate the effectiveness of our drug-likeness score, we examined how the ADME-DL model captures the progressive optimization of compounds during the drug discovery pipeline. Figure 4 illustrates the change in drug-likeness scores as the focus shifts from established approved drugs (DrugBank; Knox *et al.* 2024) to more broader, investigational compounds (ZINC-World, ZINC-Investigational), and finally to AI-generated molecules (using Guan *et al.* 2023) with minimal experimental validation.

We observe a descending trend in both median and mean drug-likeness scores as we progress toward less characterized or more speculative chemical entities. These results suggest that ADME-DL inherently captures the higher likelihood of established drugs to exhibit favorable ADME properties and aligns with the intuitive expectation that compounds further from clinical status often score lower in drug-likeness.

**Figure 5.**
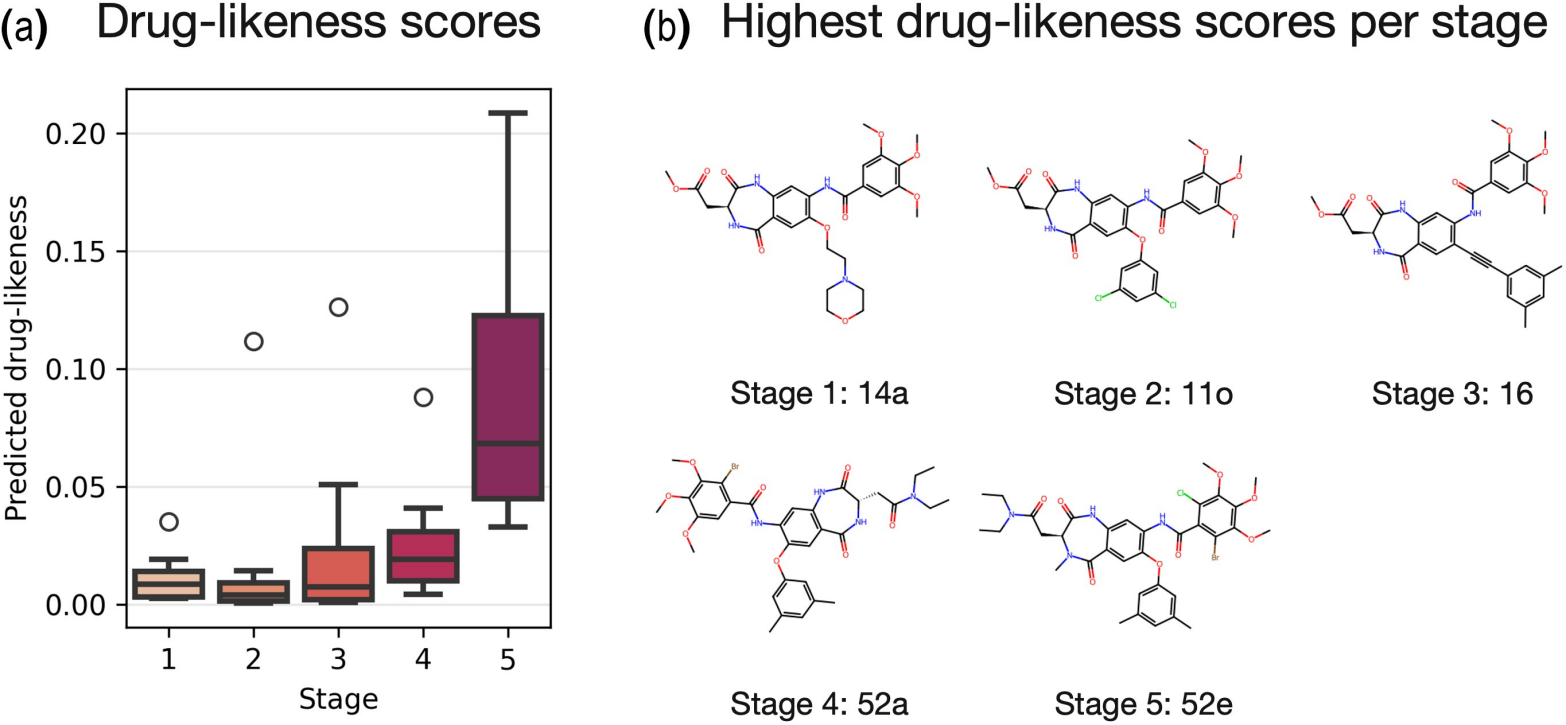
Distribution of drug-likeness score by ADME-DL on 52 compounds in a case of potential protein synthesis inhibitor discovery. The drug-likeness score improves as drug discovery steps proceed.

We further conducted a focused case study on a protein synthesis inhibitor discovery project (Yu *et al.* 2022) following the study by Gu *et al.* (2024) (Fig. 5). By dividing 52 synthesized compounds into five development stages, we observed a clear upward trend in predicted drug-likeness scores. Early-stage compounds averaged a low score (0.01), while final-stage compounds reached an average of 0.09, with some nearing 0.20—indicating a consistent upward shift in drug-likeness. This progression highlights our model’s ability to capture real-world medicinal chemistry refinement as compounds evolve toward drug-like profiles and ADME viability.

Collectively, these observations demonstrate that ADME-DL not only discriminates between known drugs and nondrugs but also mirrors the stepwise progress of rational drug design. As such, ADME-DL serves as a potential tool for guiding decisions at various stages of drug development, highlighting compounds that are more likely to succeed in downstream optimization and clinical testing.

### 3.5 Visualization of drug-like space

**Figure 6.**
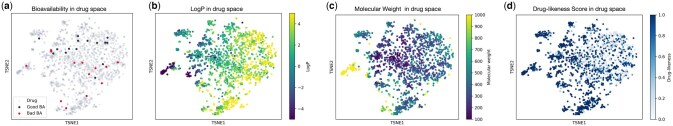
t-SNE visualization of the embedding space of ADME-DL model. Total 1964 drugs are color-coded by (a) bioavailability, (b) LogP, (c) molecular weights, and (d) drug-likeness score from ADME-DL model.

To further elucidate how sequential training reshapes molecular embeddings, we visualized the learned representation space of approved drugs using tSNE dimension reduction (Fig. 6). Figure 6a visualizes clinically annotated drugs labeled with good (blue) and poor (red) bioavailability (BA) within our learned embedding space. While the two groups are not strictly separable, we observe a noticeable improvement in local clustering structure with Sequential ADME MTL. Specifically, the clustering quality measured with silhouette score increases from 0.11 (vanilla MFM) to 0.20 (Sequential ADME MTL), indicating enhanced local differentiation between the bioavailability groups. The complete list of experimented BA-annotated drugs is provided in Supplementary Table S14.

As depicted in Fig. 6b and c, color-coding the tSNE plots by physicochemical properties (e.g. molecular weight, logP) revealed tighter grouping of molecules based on the labels. These results suggest that, despite not being explicitly taught to group molecules by these properties, our approach inherently encodes key components of traditional drug-likeness context into the learned representation. Lastly, color-coding with our models’ drug-likeness scores summarized the distribution of drug-likeness on the ADME-learned space, with the majority of them correctly showing high profiles.

## 4 Conclusion

In this work, we introduced a novel framework for ADME-informed drug-likeness prediction, distinguishing itself from traditional structure-only approaches. This approach not only resolves conflicts among ADME tasks but also enhances representational power across diverse MFMs, including both GNN and Transformer architectures. In extensive experiments on three distinct drug-likeness datasets, our model achieved consistent improvements in classification performance, which underscores the importance of modeling interdependent PK processes.

By constructing an ADME-informed embedding space, we bridged the gap between purely structural descriptors and the pharmacological behavior of compounds. Our approach is designed in a model-agnostic way, so that learned embedding representations are consistently aligned with real-world PK principles in case studies. Although our method shows strong potential for early-stage candidate screening, future work could expand its scope by integrating toxicity endpoints or exploring additional ADMET attributes, where modeling dependencies become more challenging with the inclusion of toxicity. We believe this biologically guided and data-driven MTL framework offers a promising new direction in computational drug discovery, contributing for more efficient and accurate drug development pipelines.

## Author Contributions

D.B., J.L., H.S., and S.K. conceived the study and experiments, D.B. and J.L. conducted the experiments, D.B., J.L., H.S., and S.K. analyzed the results, D.B. and H.S. drafted the manuscript, and all authors reviewed the manuscript.

## Supplementary Material

btaf259_Supplementary_Data

## Data Availability

All datasets utilized in this study are publicly available from DrugMAP (https://drugmap.idrblab.net/), ZINC20 (https://zinc20.docking.org/), PubChem (https://pubchem.ncbi.nlm.nih.gov/), ChEMBL (https://www.ebi.ac.uk/chembl/), and TDC (https://tdcommons.ai/). The source code is accessible online at https://github.com/eugenebang/ADME-DL.
